# Performance Optimization and Long-Term Strength of Basic Magnesium Sulfate Cement Prepared with Accelerated Carbonated Boron Mud

**DOI:** 10.3390/ma18184231

**Published:** 2025-09-09

**Authors:** Jiankun Li, Xiaowei Gu, Bohan Yang, Shenyu Wang, Zhihang Hu, Ziyang Hu, Xiaowei Ge

**Affiliations:** 1 Science and Technology Innovation Center of Smart Water and Resource Environment, Northeastern University, Shenyang 110819, China; 2 Liaoning Institute of Technological Innovation in Solid Waste Utilization, Shenyang 110819, China

**Keywords:** magnesium cement, carbonation, boron mud, hydration properties

## Abstract

Basic magnesium sulfate cement (BMSC) has attracted increasing attention as a low-carbon alternative to traditional Portland cement. Therefore, this study investigates the feasibility of using carbonated boron mud (CBM), an industrial solid waste, as a partial substitute for magnesium oxide (MgO) in BMSC. Prior to its incorporation into the cementitious matrix, boron mud (BM) underwent rapid carbonation treatment to improve its reactivity, microstructure compatibility, and CO_2_ sequestration potential. Experimental results from macroscopic and microscopic analyses confirmed the effectiveness of the carbonation process, showing that the carbonate ions carried by the CBM were successfully incorporated into the cementitious system. These carbonate ions reacted with MgO to form stable magnesium carbonate phases, effectively suppressing the formation of magnesium hydroxide (Mg(OH)_2_), which typically detracts from strength and stability. Compared to BMSC specimens containing untreated BM, the CBM-modified BMSC exhibited significantly improved mechanical performance and excellent volume stability. Furthermore, the carbonation pre-treatment effectively mitigated volumetric instabilities associated with rapid MgO hydration, thereby promoting a more favorable environment for the formation of the crucial 5·1·7 phase (5Mg(OH)_2_·MgSO_4_·7H_2_O). Overall, this research presents a promising strategy for producing CBM-BMSC, offering a sustainable approach to CO_2_ utilization and enhancing the volume stability of magnesium-based cements, providing a new direction for improving the sustainability of the concrete industry and advancing the development of magnesium cements.

## 1. Introduction

Boron mud (BM) is a major industrial solid waste generated during borax production, typically producing approximately 3–4 tons of residue per ton of borax manufactured [1,2,3]. This residue accounts for about 50–60% of the total mass processed in boron extraction and is predominantly disposed of in landfills, leading to significant land occupation and potential groundwater contamination [4,5,6]. Additionally, due to its fine particulate nature, BM is susceptible to wind dispersion, thereby aggravating localized air pollution issues [7,8].

Characterization studies indicate that BM primarily consists of magnesium-rich minerals, including enstatite (Mg_2_SiO_4_), magnesite (MgCO_3_), dolomite (CaMg(CO_3_)_2_), and minor quartz (SiO_2_) [9,10]. Due to this abundant magnesium content, extensive research has explored strategies for magnesium recovery from BM. For instance, Bo et al. (2017) investigated magnesium recovery using aqueous solutions pressurized with CO_2_, demonstrating preliminary feasibility despite relatively limited extraction efficiencies [11]. Similarly, Chang et al. (2023) conducted detailed studies on the kinetics of magnesium leaching using ammonium bisulfate solutions, affirming the theoretical potential for magnesium recovery [12]. Nevertheless, the practical implementation of this method remains challenging due to complex process variables. In another notable approach, Ning et al. successfully utilized waste sulfuric acid for the extraction of MgSO_4_·7H_2_O from BM, achieving extraction efficiencies exceeding 90% [13]. Despite this promising outcome, economic constraints continue to present significant barriers to industrial-scale implementation.

Considering the high magnesium content in BM, parallel research efforts have been dedicated to directly integrating BM into magnesium-based cementitious materials. Yu et al. (2021) developed magnesium phosphate cement (MPC) incorporating BM, obtaining satisfactory compressive strength but encountering long-term stability issues [14]. Further supporting the feasibility of using BM in cementitious systems, recent studies by Wang et al. (2024) and Ruan et al. (2025) have utilized chemically similar aluminosilicate wastes, such as red mud, in basic magnesium sulfate cement (BMSC) and MPC formulations, respectively [15,16]. Collectively, these efforts have provided both theoretical and experimental foundations for exploring BM’s compatibility and potential advantages within various magnesium cement formulations.

Among magnesium-based cements, BMSC has emerged as a promising alternative developed to address the limitations of magnesium oxysulfate cement (MOS), particularly in terms of durability and water resistance [17]. BMSC exhibits rapid setting characteristics, excellent fire resistance, low thermal conductivity, chemical stability, and robust mechanical properties, attributed mainly to the formation of the stable hydration product the 5·1·7 phase (5Mg(OH)_2_·MgSO_4_·7H_2_O). It is the principal strength-enhancing hydration product in BMSC. It exhibits a stable hexagonal crystal structure with a dense, interlocked needle-like morphology, fills the voids caused by brucite expansion, and is crucial for both early and long-term strength development [18,19]. Unlike traditional CaO-rich silicate cements, BMSC represents a magnesia-based cementitious system that develops strength via the hydration of reactive MgO and the formation of the 5·1·7 phase, rather than through pozzolanic reactions [20]. In the context of global efforts to reduce carbon emissions, BMSC has attracted attention due to its unique capability for active CO_2_ sequestration during hydration, offering significant environmental advantages compared to traditional Portland cement, which emits around 840 kg CO_2_ per ton produced. It has the potential of carbon-negative building materials [21,22].

Recently, rapid carbonation technology has gained prominence as an effective method to enhance both the performance and sustainability of cement-based materials [23]. Mineral carbonation, involving the reaction between CO_2_ and alkaline minerals to produce stable carbonates, has been extensively studied as a viable method for long-term carbon storage and improved material performance [24]. With respect to durability-oriented investigations, You et al. (2024) demonstrated that carbonation curing beneficially modifies the pore structure of magnesium-based cements, indicating a positive influence on durability [25]. In magnesium cement research, carbonation approaches mainly include post-curing carbonation and pre-carbonation of raw materials. Xiang et al. (2024) investigated the carbonation treatment of coal gasification ash, reporting improvements in both mechanical performance and rheological behavior of cementitious matrices [26]. Moreover, Zhang et al. (2024) examined rapid carbonation of calcium-bearing minerals to increase carbonation efficiency [27]. Post-curing carbonation, however, often suffers from limited CO_2_ penetration, as evidenced by Kashef-Haghighi (2010), who observed carbonation primarily at material surfaces [28]. In contrast, pre-carbonation of raw materials, as demonstrated by Tian et al. (2025) [29] and Gu et al. (2024) [30], effectively improves carbonation efficiency and overall reactivity in cementitious systems. Tian et al. (2025) reported significant improvements in mechanical properties by pre-carbonating titanium slag before incorporation into MPC [29]. These strategies primarily mitigate the rapid and undesired formation of magnesium hydroxide (Mg(OH)_2_), which negatively impacts material strength and durability. Through carbonation, Mg(OH)_2_ formation is inhibited, favoring instead the generation of stable magnesium carbonate phases, thus significantly enhancing the mechanical integrity and long-term performance of magnesium cement-based materials. Conversely, Gu et al. (2024) found that pre-carbonation treatment of steel slag effectively enhanced subsequent material performance in autoclaved aerated concrete formulations [30]. Consequently, pre-carbonation of raw materials has increasingly been recognized as a superior method, offering greater potential for overcoming CO_2_ diffusion limitations and achieving enhanced carbonation efficiency.

Given BM’s mineralogical profile, which is dominated by low-reactivity silicate and carbonate phases, its intrinsic hydration activity remains limited. Nonetheless, the fine particle size and magnesium-rich composition of BM make it suitable for rapid carbonation treatments [31]. Specifically, minerals such as enstatite and dolomite can readily supply Ca^2+^ and Mg^2+^ ions for carbonate formation, thereby enhancing the reactivity and carbon sequestration potential of BM [32]. Even partial carbonation can effectively introduce carbonate ions into the cementitious system, significantly improving its reactivity and environmental profile. Importantly, rapid utilization of carbonated BM in construction applications could mitigate concerns regarding long-term stability or re-carbonation [33].

To address the energy intensive and often uneconomical nature of conventional boron mud recovery routes based on calcination or acid leaching, this study proposes a cost-effective and straightforward alternative: direct rapid carbonation of boron mud (CBM) and its use as a partial MgO replacement in BMSC, thereby establishing the core novelty and practical advantage at the outset. We then comprehensively evaluate the effects of CBM on mechanical performance, volume stability, and carbonation efficiency, employing XRD, TG–DTG, FTIR, and SEM to characterize hydration processes, microstructural evolution, and strength development. Particular attention is given to the role of CBM in promoting the stable formation of the desirable 5·1·7 phase (5Mg(OH)_2_·MgSO_4_·7H_2_O), which underpins the observed performance gains. Collectively, the results demonstrate enhanced strength and stability when CBM is incorporated into BMSC and provide a scalable pathway for the sustainable utilization of boron mud, distinguishing this work from prior studies on red mud and boron mud systems and pointing to an innovative, low-carbon direction for magnesium-based cementitious materials.

## 2. Materials and Methods

### 2.1. Materials

Boron mud (BM) was supplied by Yingkou Ding’an Group (Yingkou, China) as a byproduct of borax production. Prior to use, the material was dried, ground, and passed through a 75 μm sieve. Its chemical composition (Table 1) was determined by X-ray fluorescence spectroscopy (XRF; PANalytical Axios, Almelo, The Netherlands) [34], and its mineral phases were identified via (XRD; Rigaku Ultima IV, Tokyo, Japan), revealing magnesite (MgCO_3_), dolomite (CaMg(CO_3_)_2_), and enstatite (MgSiO_3_) as the dominant constituents. These phases are known to undergo carbonation under appropriate conditions. These phases are known to undergo carbonation under appropriate conditions. Based on their low abundance and largely inert silicate/aluminosilicate character, the non-magnesian constituents in BM are unlikely to exert a measurable influence on the BMSC system; accordingly, their effects are not further discussed in this study. Light-burned magnesia (MgO) was provided by Xifeng Group (Dashiqiao, China) and was calcined at 850 °C for 2 h. The active MgO content (~67.5 wt %) was measured according to the standard MgO activity test (Section 2.2.2). Its major oxide composition is listed in Table 2 (XRF; PANalytical Axios, Almelo, Netherlands) [34]. Given their low levels and impurity nature, the minor non-magnesian oxides in the light-burned MgO are not expected to materially influence the BMSC hydration chemistry or the formation of the 5·1·7 phase; accordingly, their effects are not further discussed in this study. Magnesium sulfate heptahydrate (MgSO_4_·7H_2_O) (reagent grade, ≥98 wt %) was also supplied by Xifeng Group (Liaoning, China) and dissolved in deionized water (Qipeng Reagent Company, Liaoning, China) to prepare a 25 wt % MgSO_4_ solution. Citric acid (reagent grade, Citric Acid Monohydrate, GB/T 9855-2008, Huasheng Reagent Company, Tianjin, China) [35] was obtained from Hebei Province, China, and employed as a set-retarder to mitigate early-age cracking induced by the rapid hydration of MgO. All materials were used as received without further modification.

#### Magnesium Oxide Activity Detection Method

The hydration activity of light-burned magnesia was measured following the Chinese standard WB/T 1019-2002 [36] “Light-Fired Magnesia for Magnesite Products.” A 10 g MgO sample was placed in an oven at 105 °C for 4 h, then dried at 150 °C for an additional 2 h until a constant mass was achieved. The hydration activity (A) was calculated using:

(1) $$W=\frac{W_{2}-W_{1}}{0.45\;W_{1}}\times 100\%$$

where W_1_ is the initial dry mass of the MgO sample (g) and W_2_ is the mass after hydration (g). The factor 0.45 accounts for the mass gain per gram of MgO upon full hydration to Mg(OH)_2_.

### 2.2. Test and Characterization Methods

#### 2.2.1. Preparation of Boron Mud Carbonation

**Figure 1:**
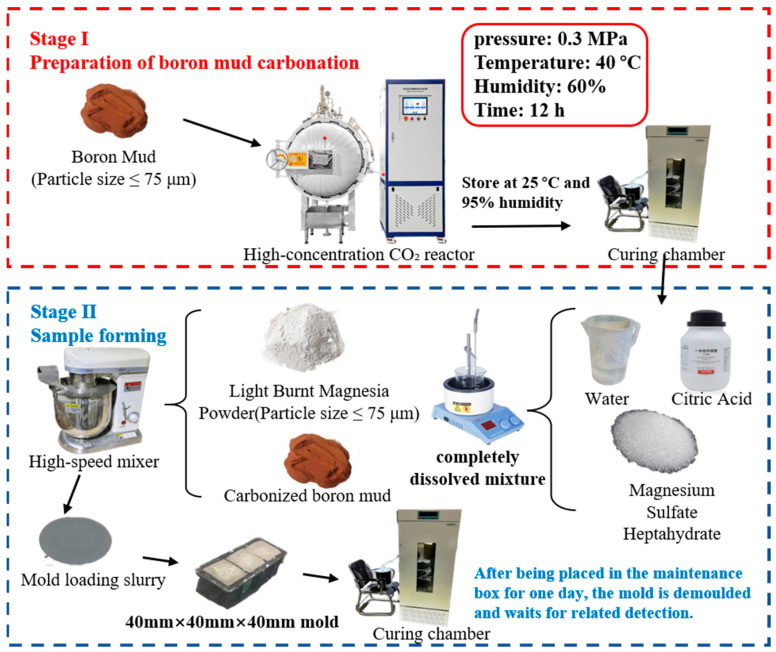
Experimental flow chart.

Carbonation of the BM was performed in a high-pressure, high-concentration CO_2_ reactor (model NELD-hpc079, NELD Intelligent Technology Co., Ltd., Beijing, China; as shown in Figure 1). Based on literature precedents, CO_2_ pressures between 0.1 and 0.5 MPa are typically employed for cementitious material carbonation [37]. In this work, the reactor pressure was fixed at 0.3 MPa to accelerate carbonation, in line with Wang et al. [38]. CO_2_ was supplied at 60 vol %, while the reactor temperature and relative humidity were held at 40 °C and 60%, respectively, throughout the process. Pressure was ramped over 20–30 min and controlled to within ±0.01 MPa to prevent rapid fluctuations that might compromise BM stability. The carbonation reaction proceeded for 12 h. A carbonation duration of 12 h was adopted because preliminary trials showed that CO_2_ uptake and carbonate-related FTIR bands approached a plateau by ~10–12 h under the present P–T conditions, with no appreciable improvements on further extension; thus, 12 h was selected as a process-efficient condition. Upon completion, the CBM was immediately transferred to a conditioning chamber maintained at 20 ± 1 °C and 95% RH for subsequent use.

#### 2.2.2. Specimen Preparation of BMSC Sample

**Figure 2:**
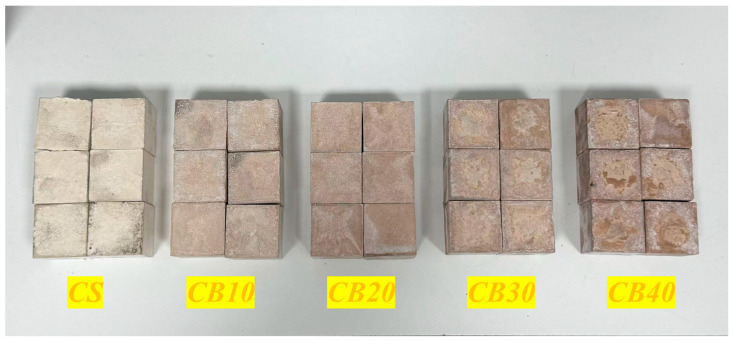
Paste specimen of CBM-BMSC samples.

Due to the absence of an official national standard for the preparation of BMSC in China, this study referred to the “General Portland Cement” standard (GB/T 175-2023) [39] and drew on extensive previous research and preliminary experiments to determine the optimal mix proportion. The chosen mass ratio for preparing the BMSC paste was MgO: MgSO_4_:H_2_O = 100:100:1. BMSC samples were fabricated using an in situ compaction procedure. First, 153.75 g of MgSO_4_·7H_2_O was mixed with 146.3 g of deionized water and heated in a water bath at 50 °C until fully dissolved. Subsequently, citric acid, accounting for 1% of the magnesium oxide mass, was added to facilitate the formation of the 5·1·7 phase. The resulting solution was transferred into a high-speed mixer (model NJ-160A, Rongjid, Shanghai, China) and stirred at a low speed of 60 r/min for 2 min to produce a homogeneous magnesium sulfate paste. Next, a total mass of 499 g of lightly burned magnesia and BM (with mass fractions of 0%, 10%, 20%, 30%, and 40%, respectively) was added to the prepared magnesium sulfate paste according to the proportions listed in Table 3. The mixture was stirred at a higher speed of 300 r/min for 2 min to obtain a uniform BMSC paste. This paste was then poured into steel molds with dimensions of 40 mm × 40 mm × 40 mm and vibrated on a shaking table for 3 min to eliminate entrapped air. Figure 2 shows a representative photograph of the CBM–BMSC paste specimens immediately after molding. After curing at ambient conditions for approximately 24 h, the samples were demolded and allowed to rest for 2 h to ensure complete surface drying. Subsequently, the demolded specimens were placed in a curing chamber maintained at 25 ± 2 °C and 60 ± 5% relative humidity. The compressive strength of the specimens was tested after curing periods of 3, 7, 28 and 90 days. To systematically evaluate the effects of BM before and after carbonation on the properties of BMSC, specimens were prepared using both raw and carbonated BM under identical fabrication and curing conditions, and their performance was assessed simultaneously at each curing age to ensure experimental consistency and comparability.

#### 2.2.3. Macroscopic Characterization of Mechanical Properties Analysis

For compressive strength testing, after completing the preparation process described in Section 2.2.2, BMSC samples were placed in a drying oven at 105 ± 5 °C until a constant weight was reached. Subsequently, compressive strength tests were conducted using a Cangzhou Longitude and Latitude 300 CS electronic servo testing machine (Cangzhou, China) in accordance with the standard GB/T 17671-2021 [40] (“Cement Mortar Strength Inspection Method (ISO)”). The specimens, measuring 40 mm × 40 mm × 40 mm, were subjected to compression testing at a loading rate of 2 mm/min, with the machine’s maximum load capacity being 300 kN. The compressive strength was evaluated at curing ages of 3, 7, 28, and 90 days. For each of the 10 groups with varying mix proportions, three replicate measurements were conducted to obtain average values for analysis.

#### 2.2.4. Microscopic Characterization

To systematically evaluate the effects of different dosages and forms of BM on the performance of BMSC, a comprehensive series of physicochemical analyses was performed, including raw material characterization, identification of hydration products, evaluation of thermal behavior, and microstructural analysis.

The transformations of RBM and CBM were analyzed by measuring pH and particle size distribution. The pH values were determined following the national standard GB/T 9724-2007 [41], using a solid-to-liquid mass ratio of 1:25 in deionized water. After equilibration, pH values were recorded using a precision pH meter (Lichen Equipment Company, Liaoning, China). For particle size analysis, samples were dried, ground to below 75 μm, and analyzed using a Malvern Mastersizer 2000 laser particle size analyzer (Malvern Instruments Ltd., Malvern, UK). The D10, D50, and D90 values were recorded to evaluate agglomeration behavior and carbonate formation. XRD was conducted to identify mineral phases present in the samples. FTIR and TG/DTG were utilized to examine changes in carbonate functional groups and CO_2_ fixation capacity. FTIR spectra were recorded in the range of 500–4000 cm^−1^ at a resolution of 4 cm^−1^. TG/DTG analyses were performed using a Netzsch STA 449 F3 thermal analyzer (NETZSCH-Gerätebau GmbH, Selb, Germany) under a nitrogen atmosphere (60 mL/min), heated from 40 °C to 900 °C at a rate of 10 °C/min.

The hydration products of BMSC were further characterized using XRD, TG/DTG, and FTIR techniques (XRD; Rigaku Ultima IV, Tokyo, Japan; TG/DTG, NETZSCH-Gerätebau GmbH, Selb, Germany and FTIR, Thermo Fisher Scientific, Madison, WI, USA.). Paste samples were vacuum-dried for 72 h, ground, and passed through a 75 μm mesh before analysis. XRD was carried out with Cu Kα radiation (λ = 0.15418 nm), using an operating voltage and current of 40 kV and 40 mA, respectively. Samples were scanned from 5° to 90° (2θ) at a step size of 0.026°. TG/DTG analyses were conducted under the same conditions described earlier, enabling assessment of phase stability and decomposition characteristics. FTIR spectra provided insights into functional group changes (e.g., C–O, O–H bonds), thereby aiding in the identification of carbonate, hydroxide, and hydrated silicate phases within hydration products.

Isothermal calorimetry was employed using a TAM Air isothermal calorimeter (TA Instruments, New Castle, USA) to investigate the influence of BM on early hydration kinetics. Freshly mixed paste samples were sealed in the calorimeter and tested at 25 °C, continuously recording heat flow rates and cumulative heat release over a period of 72 h. External disturbances and thermal fluctuations were carefully minimized during testing to ensure data reliability and accuracy.

Microstructural and compositional characterization were performed using a Zeiss scanning SEM equipped with EDS (Carl Zeiss Microscopy GmbH, Jena, Germany). Samples were gold-coated prior to analysis. SEM imaging was conducted at an accelerating voltage of 20 kV, with a resolution of 0.8 nm at 15 kV and magnifications ranging from 12 × to 1,000,000 ×. EDS spectra were acquired with a fixed acquisition time of 60 s per spectrum. Multi-point and area elemental mapping were performed to investigate element distribution patterns and interfacial bonding characteristics between BM particles and the hydration products.

## 3. Results

### 3.1. Theoretical and Actual CO_2_ Fixation Analysis of Boron Mud

The BM utilized in this study was sourced from industrial production facilities involved in the manufacture of borax and boric acid, with boron-magnesium ore serving as the primary raw material. After undergoing acid leaching treatment, most boron elements were effectively removed [42]. Phase identification by XRD (as shown in Section 2.1) revealed that the BM primarily comprises calcite, magnesite, dolomite, and enstatite, with minor quartz. Under ambient temperature and pressure conditions, these minerals typically exhibit low reactivity with CO_2_, thereby limiting substantial carbonation reactions [43]. However, the rapid carbonation reactor employed in this study provided enhanced thermodynamic and kinetic conditions—elevated pressure, increased temperature, and the presence of moisture—which collectively favored mineral carbonation reactions within the BM. According to Liu et al. [44], calcite, magnesite, and dolomite can react with CO_2_ and water under specific temperature and pressure conditions to yield their respective bicarbonates, as shown by the following equations:

CaCO_3_(s) + CO_2_(aq) + H_2_O(l) ⇌ Ca^2+^(aq) + 2 HCO_3_^−^(aq) (2)

MgCO_3_(s) + CO_2_(aq) + H_2_O(l) ⇌ Mg^2+^(aq) + 2 HCO_3_^−^(aq) (3)

CaMg(CO_3_)_2_(s) + 2 CO_2_(aq) + 2 H_2_O(l) ⇌ Ca^2+^(aq) + Mg^2+^(aq) + 4 HCO_3_^−^(aq) (4)

Although Ca(HCO_3_)_2_ and Mg(HCO_3_)_2_ are thermodynamically unstable and prone to decomposition at room temperature, making them unsuitable for long-term storage, their transient formation is sufficient for achieving immediate CO_2_ fixation in this study [45]. Therefore, the introduction of carbonate ions into BM through either chemical reactions or physical adsorption during the carbonation process is considered evidence of effective carbon fixation.

To confirm the effectiveness of carbonation, several analytical techniques including pH measurement, FTIR, particle size analysis, and TG/DTG were performed on samples of BM before and after carbonation.

**Figure 3:**
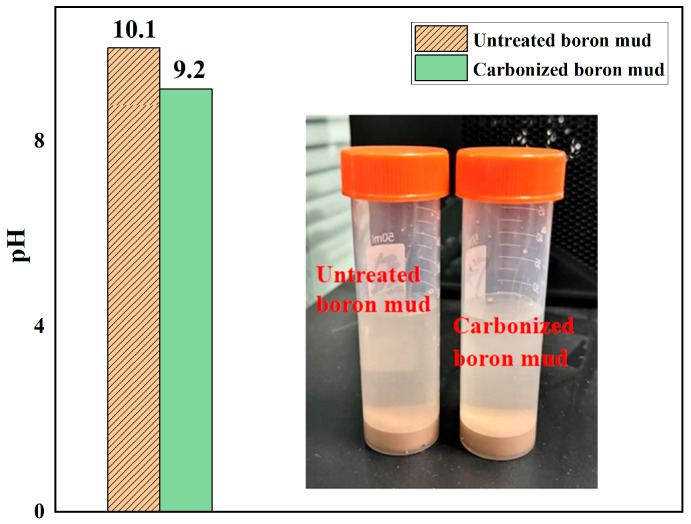
pH test results of boron mud and carbonated boron mud.

The pH test results shown in Figure 3 indicate that the pH value of RBM was approximately 10.1, while the CBM exhibited a lower pH of around 9.2. Although the carbonated sample remained alkaline, the observed reduction in pH suggests an increase in acidic species such as bicarbonate ions (HCO_3_^−^), indirectly confirming the successful incorporation of carbonate ions.

**Figure 4:**
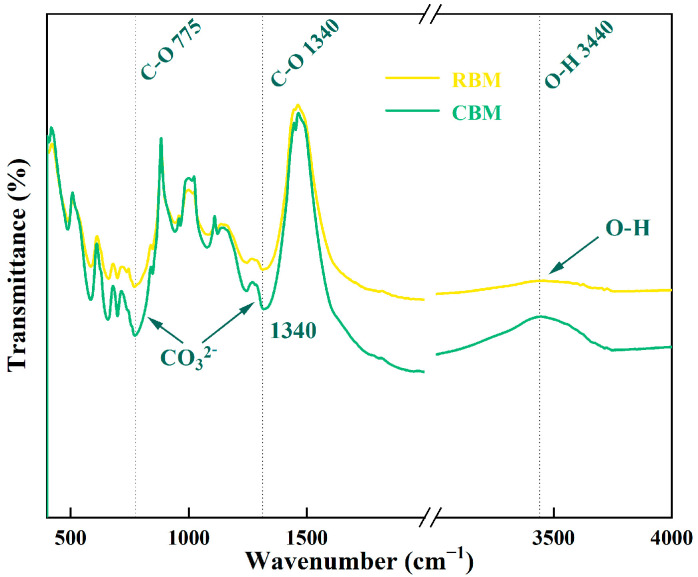
FTIR spectra of boron mud and carbonated boron mud.

Figure 4 presents the comparative FTIR spectra of BM samples before and after carbonation. Both spectra exhibited distinct absorption peaks corresponding to C–O stretching vibrations at approximately 775 cm^−1^ and 1340 cm^−1^, with increased intensities following carbonation, indicating enhanced carbonate content. Furthermore, intensified bending vibration peaks in the regions of 700–790 cm^−1^ and 1200–1380 cm^−1^ implied structural modifications in the crystal lattice. Alongside the pronounced increase in the O–H vibration peak at 3440 cm^−1^, these spectral changes collectively suggest additional carbonate formation through the reaction of CO_2_ with hydroxyl groups present in the CBM. Hence, the FTIR analysis provided convincing evidence of partial carbonation in minerals such as calcite, magnesite, and dolomite within the BM.

**Figure 5:**
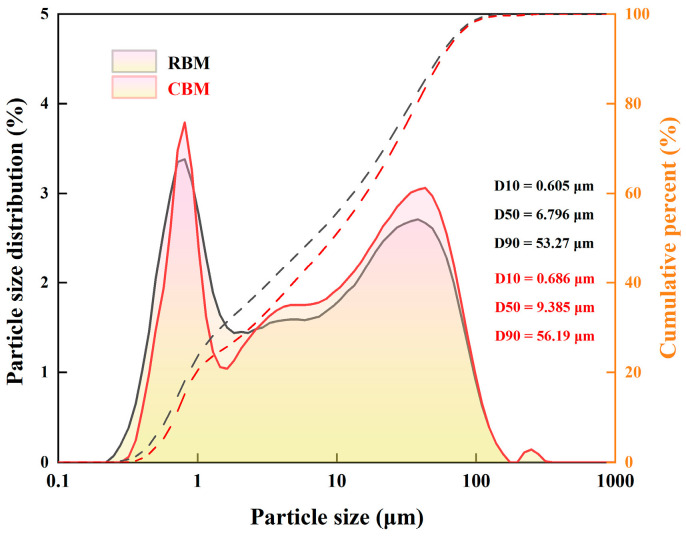
Particle size distribution of boron mud and carbonated boron mud. (Note: Solid lines = Particle size distribution (%); dashed lines = Cumulative percent (%); RBM (black), CBM (red)).

The particle size distribution analysis presented in Figure 5 further corroborates the effectiveness of carbonation. Compared with the uncarbonated sample, the CBM displayed significantly increased values of D10, D50, and D90. Specifically, the D50 value increased to 6.796 μm, and the D90 value reached 53.27 μm. This particle coarsening and enhanced agglomeration behavior can be attributed to carbonate deposition or physical CO_2_ adsorption onto particle surfaces [46]. Additionally, the resultant pore structure among particles is expected to facilitate further CO_2_ diffusion and adsorption.

**Figure 6:**
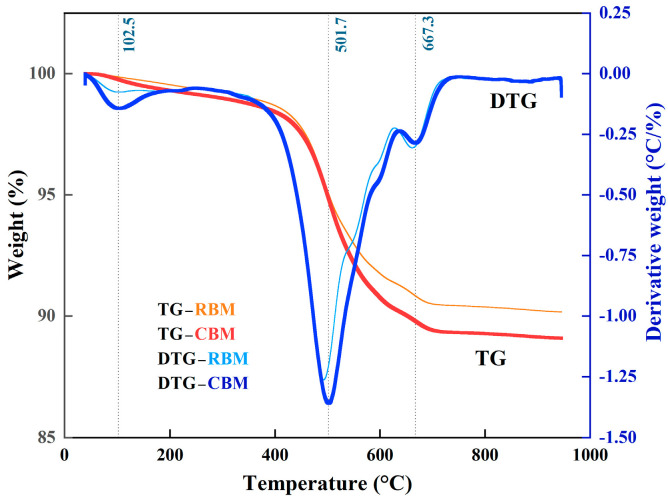
TG–DTG curve of boron mud.

Figure 6 shows the TG and DTG curves for both RBM and CBM. Each sample exhibited multi-stage mass loss characteristics. Mass loss observed below 150 °C predominantly arose from the evaporation of physically adsorbed water, as indicated by a distinct DTG peak near 102.5 °C [2]. Between 200 and 500 °C, the mass loss primarily resulted from thermal decomposition of other hydrated phases, corresponding to a DTG peak centered around 501.7 °C [4]. A pronounced difference was evident within the temperature range of 600–800 °C, where CBM displayed a significant additional mass loss accompanied by a strong DTG peak at 667.3 °C, whereas RBM exhibited minimal mass loss in this interval. This phenomenon clearly indicates the presence and decomposition of carbonate phases in the CBM, releasing CO_2_ and verifying that the carbonation process effectively immobilized a portion of CO_2_ [47].

In summary, the experimental findings demonstrate that BM possesses significant CO_2_ fixation potential under rapid carbonation conditions. The data collectively indicate that rapid carbonation facilitates further reactions between carbonate minerals such as calcite, magnesite, and dolomite with CO_2_, leading to the formation of new carbonate species. Furthermore, the results from FTIR, particle size distribution, and TG analyses suggest dual mechanisms—physical adsorption and chemical carbonation reactions—are active during CO_2_ incorporation. Thus, the outcomes of this study support the conclusion that CBM exhibits effective CO_2_ absorption capabilities and provide valuable preliminary experimental and theoretical evidence for its utilization in magnesium-based cementitious materials.

### 3.2. Effects of Uncarbonized and Carbonated Boron Mud on the Compressive Strength of BMSC

**Figure 7:**
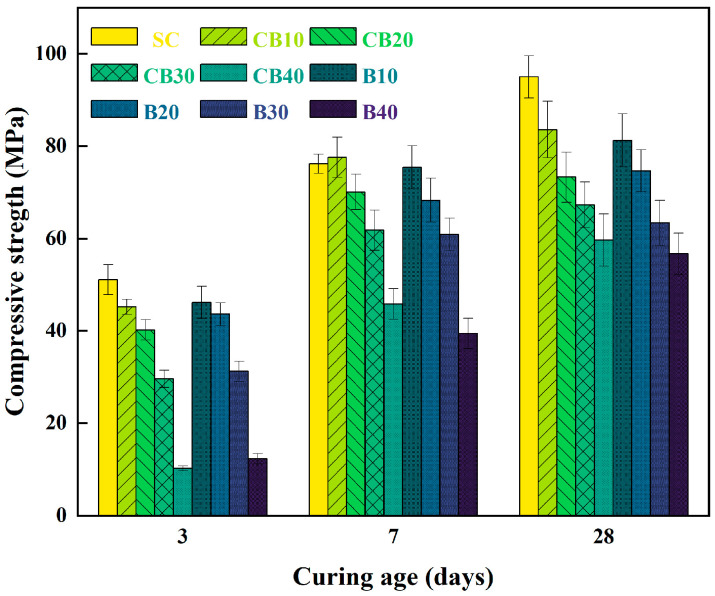
Comparison of compressive strength of BMSC samples with boron mud and carbonated boron mud.

**Figure 8:**
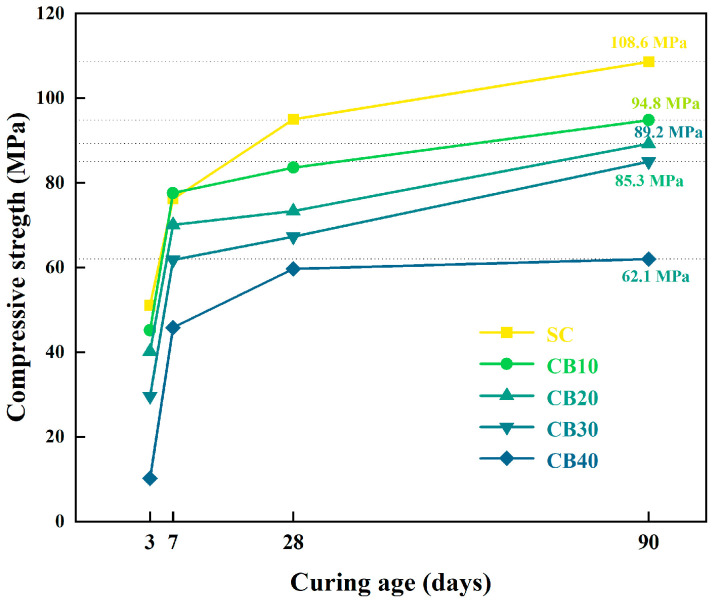
Compressive strength development of CBM–BMSC samples from 3 to 90 days.

Figure 7 and Figure 8 present the variations in compressive strength of BMSC specimens containing different proportions of RBM and CBM across curing periods of 3, 7, 28, and 90 days. Clearly, the CBM group consistently demonstrates higher compressive strength at all curing ages compared to the RBM group, accompanied by reduced error bars. This result suggests that carbonation pretreatment may contribute to strength increases and could promote a more uniform matrix distribution.

In general, an increase in CBM content correlates with a decline in early compressive strength at 3 days, with the most pronounced decrease occurring at a 40% incorporation level. As previously discussed by Wang, this phenomenon can mainly be ascribed to the retardation effect imposed by the presence of citric acid on the hydration kinetics of MgO [48]. Furthermore, due to the finer particle size of CBM, higher dosages lead to increased pore-filling and encapsulation of reactive particles, thus hindering the progression of hydration reactions [49]. Nevertheless, the CBM group displays a notable increase in compressive strength at 7 days, especially evident in the CB40 sample, which exhibits an approximately 268% strength enhancement compared to its 3-day counterpart. This observation suggests that after the initial retardation phase, hydration reactions accelerate, and the presence of CBM promotes more effective formation of compact hydration products.

Further comparison of error bars between the CBM and RBM groups highlights a substantial reduction in strength variability for the CBM group. BMSC, being an air-hardening material, typically possesses brittle hydration products, rendering it susceptible to strength fluctuations induced by external disturbances or non-uniform hydration, as reported by Wu et al. (2014) [50]. In RBM specimens, MgO hydration tends to form expansive and porous Mg(OH)_2_ phases, leading to structural instability. Conversely, carbonate ions introduced via CBM incorporation inhibit excessive formation of Mg(OH)_2_ and simultaneously facilitate crystallization of more stable magnesium carbonate phases [25]. Consequently, a denser and more uniform structural framework develops, which may enhance the stability and reproducibility of compressive strength.

Analyzing strength development trends among CBM specimens with varying dosages reveals superior long-term performance at CBM contents of 30% or higher. After the 28-day curing period, a noticeable divergence in strength emerges between the CB30 and CB40 groups, with the CB30 group exhibiting a comparatively more stable progression. This finding suggests that 30% incorporation might represent a critical dosage threshold within the cementitious system. At this dosage, CBM incorporation does not hinder the formation of the key strength-contributing 5·1·7 phase; instead, it likely promotes the crystallization and stable growth of this phase by supplying nucleation sites and creating a carbonate-rich environment, thus enhancing the mechanical performance of the cementitious matrix [51].

While the hydration process of magnesium-based cementitious materials is generally considered to reach completion around 28 days, Figure 8 reveals continued strength development up to 90 days in the CBM specimens, albeit at a slower rate. This prolonged increase in strength is likely attributed to the ongoing absorption of atmospheric CO_2_ by the specimen surfaces during curing, resulting in the formation of stable carbonate phases such as MgCO_3_ and CaCO_3_ [52]. These carbonates form a dense protective layer, further augmenting both the compressive strength and environmental durability of the cementitious matrix.

In conclusion, the partial incorporation of CBM substantially improves the compressive strength, enhances long-term stability, and contributes to a denser and more uniformly hydrated structure in the BMSC system. These results provide valuable theoretical insights supporting the potential application of CBM in high-performance magnesium-based cementitious materials. Future work will undertake a finer-grained dosage scan within 20 to 40 percent CBM to refine the optimum under the present formulation and curing conditions. Subsequent studies involving detailed phase composition analyses (XRD), SEM, and chemical bonding investigations (FTIR) will further clarify the underlying mechanisms and reinforcing effects associated with carbonation modification.

### 3.3. XRD Analysis

**Figure 9:**
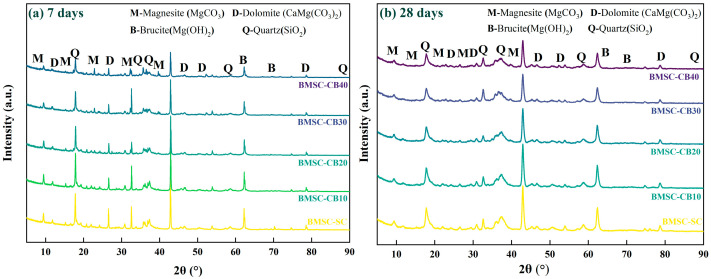
XRD patterns of CBM–BMSC samples at 7 days (**a**) and 28 days (**b**).

**Figure 10:**
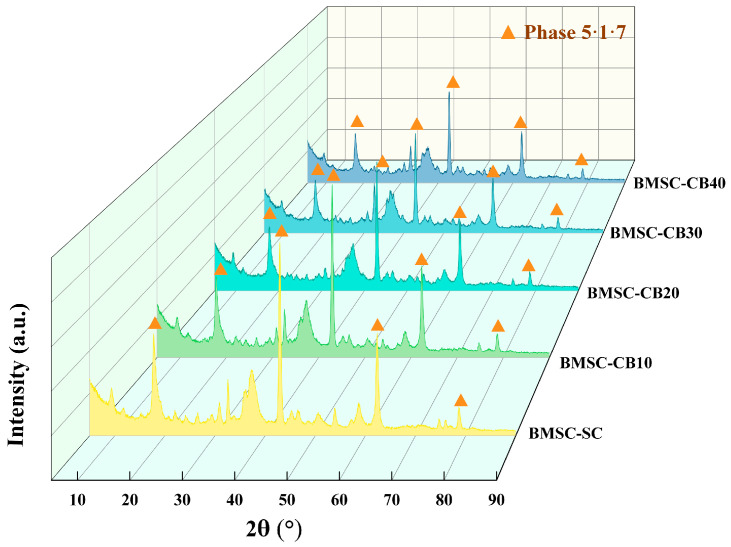
5·1·7 phase XRD pattern of CBM–BMSC samples of 28 days.

Figure 9 and Figure 10 illustrate the XRD patterns of BMSC specimens containing CBM after curing for 7 and 28 days, respectively, emphasizing the diffraction peak evolution of brucite (Mg(OH)_2_), hydromagnesite (Mg_5_(CO_3_)_4_(OH)_2_·4H_2_O), the 5·1·7 phase, and residual MgO during secondary solidification. In the CBM-BMSC specimens, the diffraction peaks corresponding to hydromagnesite markedly intensified (Figure 9), clearly indicating that carbonation pretreatment effectively enhanced CO_2_ uptake and subsequent magnesium carbonate formation. Conversely, hydromagnesite peaks also appeared in the uncarbonated BMSC samples (Figure 10), likely due to the natural carbonation of calcium carbonate and magnesium silicates inherently present in the RBM, along with residual magnesite contributions [53].

Quantitative phase analysis using Jade software (Materials Data, Inc. (ICDD), Livermore, CA, USA; https://materialsdata.com/) revealed a significant increase in the integrated intensity of hydromagnesite peaks in the carbonated samples between 7 and 28 days, demonstrating that ongoing hydration reactions actively facilitated additional magnesium carbonate formation. Notably, both brucite and the 5·1·7 phase were identified as primary hydration products within the CBM–BMSC system [54]. As reported by Chen, the 5·1·7 phase significantly contributes to the mechanical strength of magnesium sulfate cement and directly competes with brucite formation. Excessive brucite production is detrimental to cement mechanical performance [55]; thus, the primary objective of incorporating CBM in this study was to leverage the introduced carbonate ions to preferentially react with MgO, facilitating magnesium carbonate formation and concurrently suppressing brucite generation.

As depicted in Figure 9, the diffraction intensity of brucite peaks remained relatively constant from 7 to 28 days, while hydromagnesite peaks showed a marked enhancement, aligning closely with the observed strength development trend. Further comparative analysis between carbonated and uncarbonated specimens at 28 days demonstrated that the intensities of all major hydration product peaks decreased with increasing CBM or RBM content (from 0% to 40%). Nevertheless, at a 30% replacement level, CBM samples exhibited a notably stronger hydromagnesite peak, explicitly confirming the beneficial role of the carbonation pretreatment. A semi-quantitative comparison of integrated peak intensities shows that at 30% CBM the brucite signal reaches its minimum, while the 5·1·7 phase remains at a comparable level without marked reduction. This pattern indicates that CBM suppresses the strength-adverse brucite while maintaining the strength-bearing 5·1·7, which predicts an optimal and stable strength response near 30% CBM, with a clear decline only when the dosage increases to 40%.

Quantitative analysis with Jade software further indicated that uncarbonated specimens experienced an approximate 3.7% increase in brucite content, accompanied by a decrease in residual MgO content and a slight elevation in the 5·1·7 phase proportion. According to Xu et al., brucite formation is an exothermic reaction that induces micro-porosity, thereby creating favorable nucleation sites for the subsequent growth of the 5·1·7 phase. A weak positive correlation between these phases is also observable in the XRD patterns [56]. However, given brucite’s inherent chemical instability, its presence can lead to long-term fluctuations in strength performance, presenting a potential risk for industrial-scale applications.

### 3.4. TG Analysis

**Figure 11:**
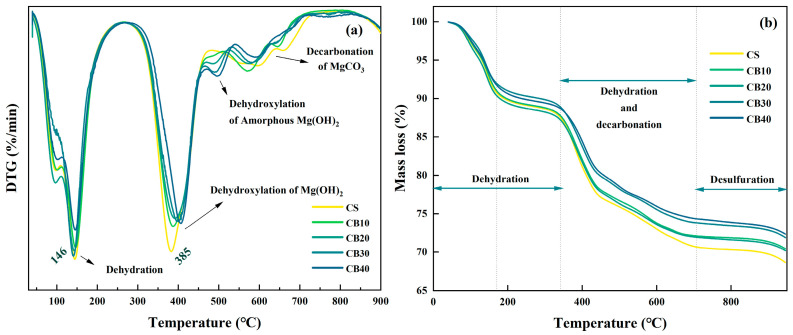
TG (**a**) and DTG (**b**) curves of CBM−BMSC samples at 28 days.

To further elucidate the influence of CBM incorporation on the hydration characteristics of the BMSC system, TG and DTG analyses were performed on samples cured for 28 days. As illustrated in Figure 11, all specimens exhibited distinct multi-stage thermal decomposition patterns. An evident endothermic peak occurred at approximately 385 °C, primarily corresponding to the dehydroxylation of Mg(OH)_2_, aligning well with previously documented observations [57]. Upon further temperature elevation to the range of 550–750 °C, substantial mass losses accompanied by distinct DTG peaks were recorded, reflecting the decomposition of carbonate-bearing hydration phases, possibly accompanied by partial desulfation of sulfate phases [58].

Comparative analyses of thermal decomposition behaviors among samples with varying CBM dosages indicated a progressive reduction in the intensity of the DTG peak at 385 °C as the CBM content increased, suggesting a corresponding decrease in the relative amount of Mg(OH)_2_ formed. This phenomenon can be attributed to the abundant carbonate ions (CO_3_^2−^) carried by CBM, which preferentially react with Mg^2+^ ions in the cementitious system, leading to the formation of stable magnesium carbonate phases, thus effectively inhibiting excessive brucite production [59]. Simultaneously, the mass losses observed in the high-temperature region (550–750 °C) were significantly more pronounced in CB30 and CB40 samples, indicating increased formation of carbonate-rich phases at higher CBM incorporation levels [60]. This result further confirms the robust carbonation efficiency of CBM and corresponds closely with the enhanced absorption bands at 1459 cm^−1^ (C–O symmetric stretching) and 789 cm^−1^ (C–O bending) detected in FTIR analyses, indirectly verifying the active involvement of CBM in the generation of carbonate species. Consistently, the DTG feature near 385 °C, which serves as a brucite proxy, is lowest at 30% CBM, whereas the mass loss from 550 to 750 °C, a magnesium carbonate proxy, remains pronounced. This combination of reduced brucite together with sustained development of carbonate and the 5·1·7 phase aligns with the observed strength trend, predicting the most stable compressive strength at 30% CBM and a noticeable decline at 40% attributable to imbalance and dilution effects.

The TG–DTG analysis provides insightful microstructural evidence supporting the mechanical property improvements previously observed. The addition of CBM not only mitigates excessive formation of the structurally weaker Mg(OH)_2_ phase but also facilitates the generation of denser and more stable magnesium carbonate phases, consequently improving the overall microstructural density and stability of the cement matrix. Such phase transformation fosters the development of a more stable 5·1·7 phase structure, which significantly enhances compressive strength and long-term durability [20]. Particularly noteworthy is the CB30 formulation, which exhibits an optimal combination of increased carbonation products and a well-developed hydration structure, indicating an ideal balance between mechanical properties and chemical stability at this CBM dosage.

Moreover, comparison of the residual mass at the completion of the TG analyses demonstrated that samples with higher CBM contents generally possessed lower final residues than the control sample, suggesting that CBM does not simply function as an inert filler but actively participates in cementitious reactions, exhibiting measurable reactivity. Such reactivity likely results from an analogous pozzolanic effect and potential nucleation-promotion activity, which could enhance the progression and continuity of hydration reactions, thereby contributing positively to microstructural densification and overall hydration advancement [61]. Collectively, these results substantiate that CBM demonstrates both advantageous reactivity and efficient carbonation capability within the BMSC system, highlighting its promising potential as a supplementary cementitious material.

### 3.5. FTIR Analysis

**Figure 12:**
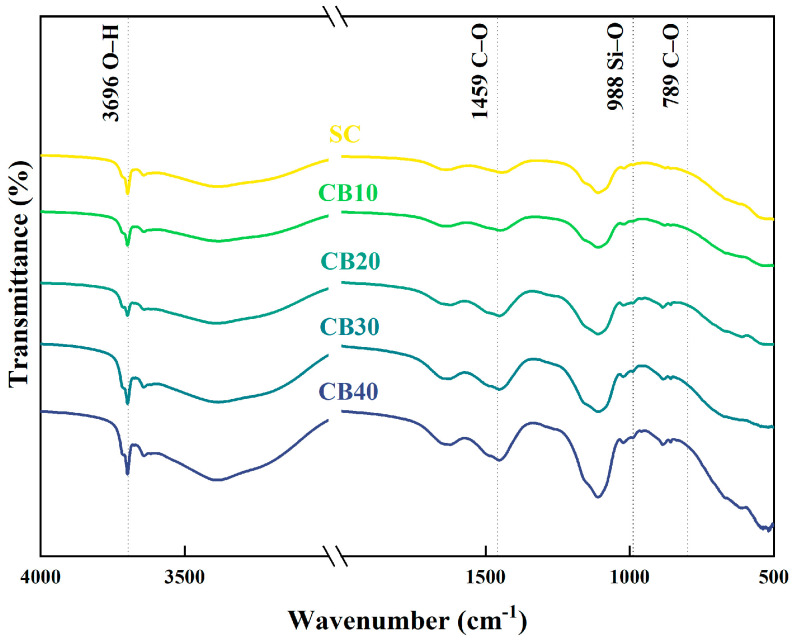
FTIR spectra of CBM–BMSC samples at 28 days.

To further elucidate the effects of CBM incorporation on the structural characteristics of hydration products in BMSC, FTIR analysis was conducted on specimens with varying CBM contents after curing for 28 days. The results, including the control group for comparative purposes, are illustrated in Figure 12.

As observed from the FTIR spectra, the characteristic C–O absorption peaks located at 1459 cm^−1^ and 789 cm^−1^ gradually intensified with increasing CBM dosage, particularly evident in samples CB30 and CB40. This enhancement signifies that carbonate ions introduced by CBM actively participated in the hydration reactions, thereby facilitating the formation of stable carbonate phases [62]. These results are consistent with the increased intensity of carbonate crystal peaks identified through XRD analysis, further validating that CBM effectively serves as a source of carbonate ions, prompting the precipitation of carbonate-bearing hydration products. Consequently, these products contribute positively to the development of a denser and more structurally stable cementitious matrix.

Conversely, the O–H stretching vibration peak appearing at 3696 cm^−1^ gradually weakened with increasing CBM incorporation, indicating a reduction in hydroxyl group content. This suggests the formation of Mg(OH)_2_ during hydration was effectively suppressed. Given that Mg(OH)_2_ typically exhibits a loosely bonded and expansive structure, its reduced formation significantly benefits the microstructural stability and volumetric integrity of cement pastes at early ages [63]. This phenomenon is consistent with previously noted mechanical strength fluctuations and pronounced variability in the RBM group, providing additional support for the superior and more stable mechanical performance of CBM-modified specimens.

Overall, the FTIR results are consistent with the observations from the mechanical and XRD analyses, suggesting that CBM incorporation may facilitate the formation of carbonate-type hydration products, potentially suppress the generation of less stable phases such as Mg(OH)_2_, and possibly contribute to a more uniform and complete hydration product network. From the perspective of functional group changes, these findings help elucidate the possible microstructural mechanisms by which carbonated boron mud could influence the structural characteristics and performance of the BMSC system, providing additional support to the interpretations discussed previously.

### 3.6. Hydration Heat Analysis

**Figure 13:**
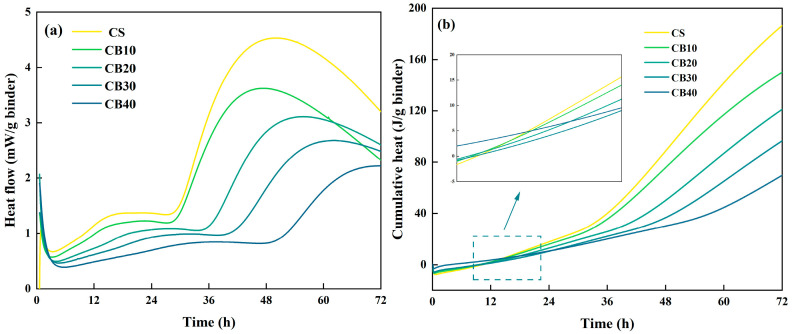
Heat flow curves (**a**) and cumulative heat release curves (**b**) of CBM–BMSC samples over 72 h.

The hydration heat evolution of BMSC pastes containing CBM was investigated through monitoring heat flow and cumulative heat release profiles over 72 h (Figure 13a,b). Notably, all specimens displayed a distinct secondary exothermic peak after approximately 24 h, contrasting markedly with the single-peak exothermic behavior typically observed in ordinary Portland cement systems [64].

In magnesium-based cement systems, the initial exothermic peak usually corresponds to the rapid reaction between magnesium oxide (MgO) and water, leading to brucite (Mg(OH)_2_) formation. However, the introduction of citric acid as a hydration retarder in this study (based on the crack-mitigation approach proposed by Wang et al.) significantly alters this conventional hydration pattern. During the initial stage, only a minor fraction of MgO reacts with water, resulting in a brief initial heat release (Figure 13a), while subsequent hydration processes are effectively regulated through the chelation action of citric acid [65]. Although this retardation delays the overall hydration reaction, it does not completely inhibit brucite formation. Importantly, the XRD analysis indicated an increased formation of the 5·1·7 phase. This observed enhancement may be attributed to the microporous structure formed by brucite precipitation, potentially providing favorable nucleation sites for the subsequent growth of the 5·1·7 phase.

The secondary heat release commenced around 24 h and extended up to 60–70 h, with the control sample reaching a maximum heat flow of 4.6 mW/g binder. Importantly, CBM incorporation delayed the onset of the secondary exothermic peak in a dose-dependent manner. Notably, the CB40 sample exhibited its secondary exothermic peak as late as 50 h, with heat flow continuing to increase even after 70 h. This delayed exothermic response can be attributed to a dual synergistic mechanism: first, the presence of CBM particles physically obstructs direct contact between MgO particles and water, thereby retarding brucite formation; second, carbonate ions released from CBM preferentially react with MgO, favoring the formation of hydrated magnesium carbonate phases and guiding the hydration pathway toward the formation of the structurally advantageous 5·1·7 phase [66]. As clearly illustrated in Figure 13b, cumulative heat release systematically decreased with increasing CBM content, aligning closely with this hypothesized reaction modulation mechanism [26]. Specifically, higher CBM contents led to suppressed overall heat release through two main effects: physical hindrance of direct MgO-water contact, and redirection of chemical equilibria toward carbonate-rich, lower-enthalpy hydration reactions. The initial low-intensity exotherm is assigned to early MgO hydration toward brucite under Mg^2+^-citrate complexation, whereas the second, dominant peak corresponds to resumed hydration and precipitation of the 5·1·7 phase together with carbonate-bearing products supplied by CBM. The rightward shift in the second peak with increasing CBM and the concurrent decrease of 72 h cumulative heat support a combined physical-hindrance and carbonate-competition/nucleation mechanism, consistent with XRD/FTIR/TG evidence.

Comparative analysis further revealed that both the control and CB10 specimens displayed a “rapid heat release–sharp decline” profile (Figure 13a), a feature typically associated with microcracking risks due to abrupt crystallization of the 5·1·7 phase [67]. In contrast, CBM-modified pastes exhibited moderated heat flow rates and prolonged hydration periods, correlating with the enhanced mechanical stability observed experimentally [68]. These observations align well with the XRD results, supporting the conclusion that an appropriate CBM dosage—particularly within the 30–40% range—can optimize hydration product distribution and control the formation kinetics of the 5·1·7 phase, thereby significantly enhancing long-term strength development.

### 3.7. SEM Analysis

**Figure 14:**
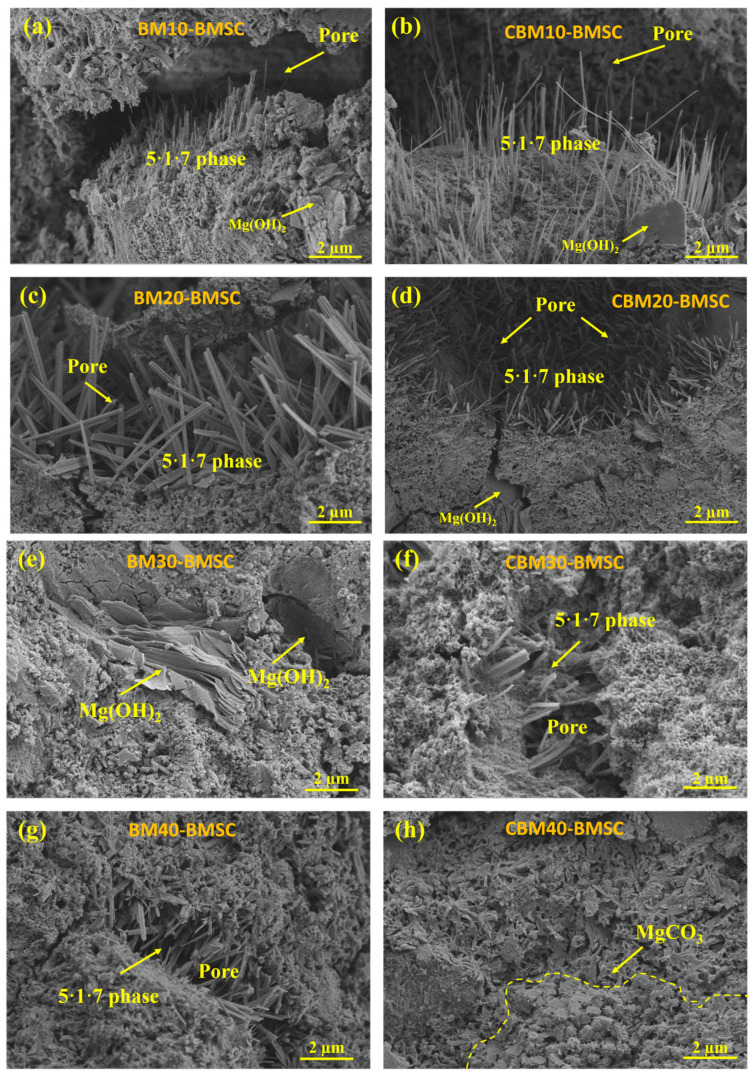
SEM images of the core cross-sections of 28-day RBM and CBM magnesium cement samples: (**a**) RBM10-BMSC, (**b**) CBM10-BMSC, (**c**) RBM20-BMSC, (**d**) CBM20-BMSC, (**e**) RBM30-BMSC, (**f**) CBM30-BMSC, (**g**) RBM40-BMSC, (**h**) CBM40-BMSC.

**Figure 15:**
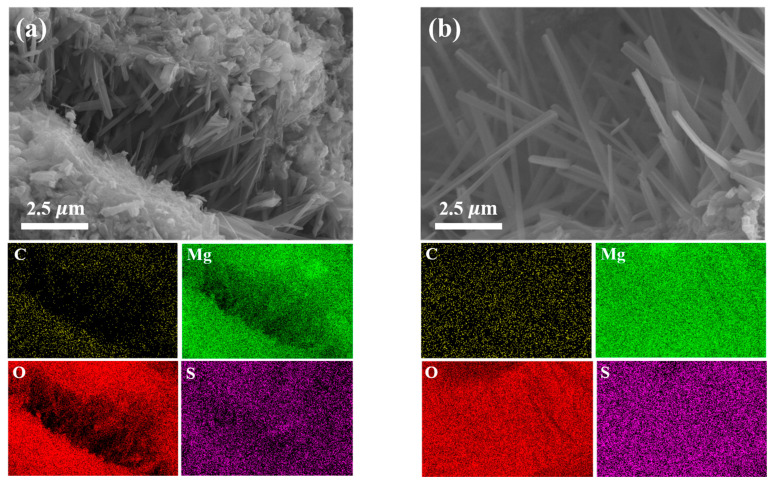
SEM-EDS cross-sectional elemental mapping of 28-day RBM and CBM magnesium cement samples: (**a**) RBM30-BMSC, (**b**) CBM30-BMSC.

**Figure 16:**
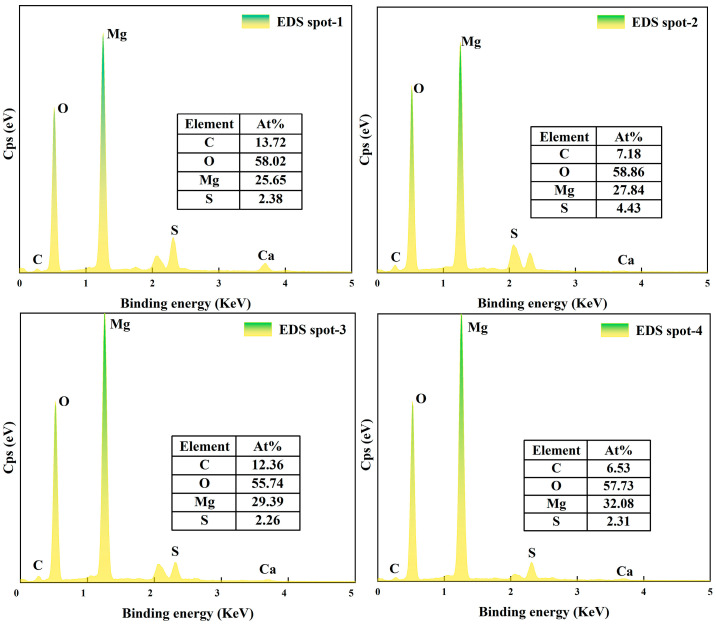
EDS elemental composition of 28-day CBM and RBM magnesium cement samples. EDS spot 1, 3-RBM30-BMSC; EDS spot 2, 4-CBM30-BMSC.

To further clarify the effect of CBM on the microstructural evolution within the BMSC system, SEM was employed to observe the morphology of inner-core sections of specimens cured for 28 days. Samples containing CBM dosages ranging from 10% to 40%, along with control samples containing RBM, were systematically examined (Figure 14). Additionally, EDS analyses were conducted on specimens containing 30% CBM and RBM (Figure 15 and Figure 16**)** to further elucidate the microstructural mechanisms associated with CBM incorporation during hydration and carbonation processes.

As depicted in Figure 14a–h, the characteristic hydration product of BMSC—the 5·1·7 phase—was consistently observed in all specimens, predominantly occupying pore spaces and exhibiting an interlaced, needle-like morphology. The formation of the 5·1·7 phase typically coincides with magnesium hydroxide (Mg(OH)_2_) precipitation during hydration [69]. Owing to its layered structure, the growth of Mg(OH)_2_ frequently introduces microporosity, thereby creating spatial regions conducive to the nucleation and subsequent growth of the 5·1·7 phase [70]. In both the 10% CBM and RBM samples, clearly defined needle-shaped 5·1·7 structures were observed alongside hexagonal, plate-like Mg(OH)_2_ crystals exhibiting a loosely bonded “book-stacked” appearance. The loose stacking and weak interlayer cohesion of Mg(OH)_2_ crystals imply structural instability, thus limiting their positive contributions to the overall mechanical integrity of the cementitious matrix. These observations correspond well with early-stage mechanical strength fluctuations typically observed in BMSC systems and reinforce the detrimental impact associated with excessive Mg(OH)_2_ formation reported in prior studies [71]. From a mechanistic perspective, the carbonate species introduced by CBM redirect the Mg^2+^ reaction pathway toward magnesium carbonates, which suppresses excess Mg(OH)_2_, a phase associated with lamellar growth, pore formation, and microcracking. The resulting fine carbonate precipitates also act as heterogeneous nuclei, improving the spatial continuity of the 5·1·7 network and refining the interfacial transition zones. As shown below, this mechanism explains why approximately 30% CBM achieves an optimal balance: brucite plates are visibly reduced while the 5·1·7 intergrowth remains well developed, yielding a more compact morphology with fewer defects.

Upon increasing the CBM content to 20%, noticeable voids and microcracks emerged around Mg(OH)_2_ crystals in both the CBM and RBM groups. These structural defects likely resulted from volumetric expansion and the loosely packed lamellar configuration of Mg(OH)_2_, thereby negatively affecting the system’s compactness and integrity [27]. Although the 5·1·7 phase continued forming within the existing pores, the growth pattern deviated from the idealized hexagonal interlocking arrangement, leading to irregular, needle-like crystals—suggesting a more unstable and less optimized hydration environment. Such Mg(OH)_2_-induced voids and cracking phenomena are commonly reported in traditional BMSC systems, which inhibit the orderly growth and network formation of the 5·1·7 phase [72,73]. These brucite-induced voids and lamellar discontinuities provide a contrast to CBM-rich mixtures, where carbonate-assisted nucleation and brucite suppression progressively densify the matrix.

In contrast, micrographs at a CBM replacement level of 30% show markedly fewer brucite plates together with compact intergrowths of the 5·1·7 phase and finely dispersed carbonate domains that often display rhombohedral or nano-scale aggregates with microporous textures [73]. These features are consistent with carbonate-assisted heterogeneous nucleation and a hydration environment in which the formation of excess brucite is effectively moderated. The resulting improvement in the spatial continuity of the 5·1·7 network and the refinement of interfacial transition zones support a denser matrix and help rationalize the observed stability of mechanical performance near this dosage.

At a CBM replacement level of 40%, carbonate features remain evident; however, partial dilution of reactive MgO, occasional agglomeration or unreacted residues, and a less continuous 5·1·7 framework begin to appear. These effects likely offset the benefits of additional carbonate and explain the diminishing returns in morphology and strength at higher dosage. This interpretation is in agreement with the phase indicators from XRD and TG, where brucite is minimized near 30% CBM without a pronounced reduction in the 5·1·7 signal, while carbonate-related thermal events remain sustained.

A detailed comparative evaluation of SEM images from the 30% CBM and RBM samples further reinforces these conclusions. The RBM sample displayed loosely stacked lamellar Mg(OH)_2_ structures accompanied by significant porosity, whereas the CBM sample exhibited denser surface morphology enriched with discernible porous rhombohedral carbonate crystals. These morphological differences explicitly confirm the active role of CO_3_^2−^ ions introduced by CBM, which enhances carbonate formation, suppresses undesirable Mg(OH)_2_ generation, and mitigates its associated structural drawbacks [74]. Consequently, these findings verify the beneficial influence of CBM in directing hydration pathways and optimizing the microstructural characteristics of BMSC systems.

Figure 15 presents the EDS elemental mapping images for the 30% CBM and RBM samples. From the elemental distribution perspective, the CBM-containing samples exhibited significantly increased carbon (C) signals on their surfaces, indicative of more abundant carbonate phase formation. This observation clearly demonstrates the effectiveness of CBM in supplying CO_3_^2−^ ions during the hydration process [75]. Apart from carbon, the distributions of oxygen (O) and magnesium (Mg) were relatively uniform between the two groups, confirming that the predominant hydration products remain magnesium-based. Thus, the differences in carbon content primarily reflect CBM’s regulatory effect on the formation and distribution of specific hydration phases.

Figure 16 presents the elemental compositions obtained via EDS spot analysis at selected regions on the 30% CBM and RBM samples, providing quantitative insights into variations in carbon content. The analyses revealed consistently higher carbon contents across all tested points in the CBM group compared to the RBM group, further affirming CBM’s superior capability in supplying CO_3_^2−^ ions. These results imply that CBM actively participates in the hydration reaction and subsequent carbonate phase formation, thus demonstrating its enhanced reactivity and functionality from a microstructural perspective. Consequently, these findings validate CBM’s potential as a valuable functional additive within magnesium-based cementitious systems [76]. Notably, at 30% CBM, the relative amount of brucite observed by SEM/EDS is lower. This agrees with the attenuation of the DTG signal near 385 °C, which marks brucite dihydroxylation, and with the sustained mass loss between 550 and 750 °C that indicates magnesium carbonate decomposition. It is also consistent with the XRD patterns, in which brucite is minimized while the 5·1·7 reflection does not show a pronounced decrease. Taken together, the evidence suggests that suppression of brucite, rather than a simple increase in carbonate content, governs the optimal morphology and strength near 30% CBM. At 40% CBM, dilution of reactive MgO and local interfacial discontinuities likely offset the benefits of additional carbonate, leading to diminishing returns. Moreover, fine capillary pores and hairline microcracks are discernible in the SEM micrographs. These defects may affect long-term durability and transport behavior under aggressive exposure. As performance in acidic or other corrosive environments was not assessed, we report these as cautious indications and identify targeted durability testing as a priority for future work.

In summary, SEM and EDS analyses collectively highlight the beneficial role of CBM in enhancing the microstructure of the BMSC system through promoting stable carbonate phase formation and effectively suppressing detrimental Mg(OH)_2_ generation. This structural enhancement significantly improves the compactness, homogeneity, and stability of the cementitious matrix. These microstructural insights, in combination with previously reported mechanical properties and thermogravimetric analysis, strongly support the multifunctional enhancement mechanism facilitated by CBM incorporation in BMSC systems.

## 4. Conclusions

In this study, CBM, prepared via rapid carbonation treatment of borax industrial solid waste, was utilized as a partial replacement for magnesium oxide (MgO) in BMSC. A comprehensive analysis—including hydration kinetics, phase composition, microstructural evolution, and mechanical performance—was performed to investigate the potential improvements imparted by CBM incorporation. The primary conclusions of this research are as follows:

1. Rapid carbonation treatment improved the reactivity of boron mud to some extent. Originally low-reactivity magnesium-rich minerals, such as magnesite, dolomite, and enstatite, underwent partial transformation into potentially reactive carbonate-containing phases, as suggested by XRD, FTIR, and TG–DTG analyses. These analytical results consistently indicated an increased carbonate content, supporting the occurrence of in situ CO_2_ fixation in the carbonated boron mud.
2. The incorporation of CBM appeared to influence the phase development within the BMSC system. The carbonate ions present in CBM possibly interacted preferentially with MgO, likely reducing the formation of excessive magnesium hydroxide (Mg(OH)_2_)—a phase known to negatively influence mechanical properties and volumetric stability. This alteration in hydration pathways may have facilitated the formation of relatively stable hydromagnesite and the structurally beneficial 5·1·7 phase (5Mg(OH)_2_·MgSO_4_·7H_2_O), as evidenced by quantitative XRD and SEM-EDS characterization.
3. CBM-modified specimens exhibited improved mechanical properties compared with those incorporating RBM, with an optimal mechanical performance observed at a CBM dosage of approximately 30%. This enhancement in compressive strength could be attributed to a potentially refined microstructure and more regulated hydration kinetics. Calorimetric analyses suggested delayed and moderated heat release behavior, which may have contributed to reduced early-age microcracking risks, thereby possibly supporting long-term durability.
4. SEM indicated that CBM incorporation resulted in a relatively denser and more homogeneous cementitious matrix, characterized by reduced porosity and potentially enhanced interfacial bonding. Elemental distribution analyses also provided supporting evidence for increased formation of stable carbonate phases, correlating well with the observed mechanical improvements in CBM-modified specimens.
5. The inclusion of CBM introduced an opportunity for in situ CO_2_ sequestration within the BMSC system. By integrating solid waste recycling with potential carbon fixation, the proposed CBM–BMSC system may not only enhance the material properties but could also contribute positively to environmental sustainability and resource efficiency.

In summary, this research highlights rapid carbonation as a potentially effective pretreatment strategy for enhancing both the performance and environmental profile of magnesium-based cementitious materials. The CBM–BMSC system investigated here represents a feasible approach toward developing sustainable construction materials through effective utilization of industrial solid waste. Future research focusing on long-term durability, comprehensive environmental assessments, and scalability for practical applications is recommended to further explore and validate the viability of this sustainable material. In addition, subsequent studies will consider the potential effects of alternative acidic and alkaline environments, as well as durability under aggressive exposures, to more comprehensively evaluate performance. We also plan to explore practical applications of CBM–BMSC, particularly in precast products and non-structural components, where the advantages of rapid setting can be most effectively utilized.

## Figures and Tables

**Table 1:** Chemical composition of BM [34].

Component	MgO	SiO_2_	Fe_2_O_3_	Al_2_O_3_	P_2_O_5_
Content (wt %)	73.51%	13.62%	10.2%	2.44%	0.23%

**Table 2:** Chemical composition of lightly burned magnesium oxide [34].

Component	MgO	CaO	SiO_2_	Fe_2_O_3_
Content (wt %)	83.3%	1.7%	6.34%	0.42%

**Table 3:** The mix proportion of BMSC specimens.

Sample Types	Boron Mud (g)	MgO (g)	MgSO_4_ (g)	Citric Acid (g)	H_2_O (g)
CS-BMSC	-	625 g	191.875 g	6.25 g	193.75 g
CBM10-BMSC	62.5 g	562.5 g	-	-	-
CBM20-BMSC	125 g	500 g	-	-	-
CBM30-BMSC	187.5 g	437.5 g	-	-	-
CBM40-BMSC	250 g	375 g	-	-	-
BM10-BMSC	62.5 g	562.5 g	-	-	-
BM20-BMSC	125 g	500 g	-	-	-
BM30-BMSC	187.5 g	437.5 g	-	-	-
BM40-BMSC	250 g	375 g	-	-	-

(Note: CS–Control Sample, CBM–Carbonized boron mud, BM–Untreated boron mud, BM10-BM40–Boron mud replacement level ranging from 10% to 40%).

## Data Availability

The original contributions presented in this study are included in the article. Further inquiries can be directed to the corresponding author.

## Abbreviations

BM	Untreated boron mud
CBM	Carbonized boron mud
BMSC	Basic magnesium sulfate cement
CS	Control sample
MOS	magnesium oxysulfate cement
DTG	Derivative thermogravimetry analysis
SEM	Scanning electron microscope
EDS	Energy-dispersive X-ray spectroscopy
FTIR	Fourier transform infrared spectroscopy
TG	Thermogravimetry analysis
MPA	Most probable pore diameter
XRD	X-ray diffractometer
XRF	X-ray fluorescence
MPC	Magnesium phosphate cement
